# Supplementary material for: Benchmarking biochemical networks generated by large language models
Source: eLife. 2026 Jul 31;15:RP109709. doi: 10.7554/eLife.109709 (PMC13427342; doi:10.7554/eLife.109709)
Supplement: Peer review history

# Editor report: eLife Assessment

**DOI:** 10.7554/eLife.109709.3.sa0

**Authors:** Martin Graña

**Affiliations:** Institut Pasteur de Montevideo Uruguay

**Keywords:** Convincing, Important

The authors propose a pipeline using large language models (LLMs) to benchmark experimentally well validated computational models of signaling networks. The findings are **important**, with available methods that guide testing future models and find new molecular interactions. Furthermore, the work shows how general-purpose LLMs can generate up to 91% of reactions of a bacterial metabolic network. The support is **convincing** and offers a number of performance metrics.

# Referee report: Reviewer #1 (Public review)

**DOI:** 10.7554/eLife.109709.3.sa1

[Editors' note: this version has been assessed by the Reviewing Editor without further input from the original reviewers. The authors did an excellent job with their resubmission, politely and elegantly answering the comments from the reviewers.]

Summary:

Large language models (LLMs) have been developed rapidly in recent years and are already contributing to progress across scientific fields. The manuscript tries to address a specific question: whether LLMs can accurately infer signaling networks from gene lists.

Strengths:

The manuscript raises a good question: whether current LLMs can accurately generate signaling networks from gene lists.

# Referee report: Reviewer #2 (Public review)

**DOI:** 10.7554/eLife.109709.3.sa2

Summary:

The authors evaluate whether commonly used LLMs (ChatGPT, Claude and Gemini) can reconstruct signalling networks and predict effects of network perturbations, and propose a pipeline for benchmarking future models. Across three phenotypes (hypertrophy, fibroblast signalling, and mechanosignalling), LLMs capture upstream ligand-receptor interactions and conserved crosstalk but fail to recover downstream transcriptional programmes. Logic-based simulations show that LLM-derived networks underperform compared to manually curated models. The authors also propose that their pipeline can be used for benchmarking future models aimed at reconstructing signalling networks.

Strengths:

The authors compare the outcomes from three LLMs with three manually curated and validated models. Additionally, they have investigated gene network reconstruction in the context of three distinct phenotypes. Using logic-based modelling, the authors assessed how LLM-derived networks predict perturbation effects, providing functional validation beyond network overlap.

Weaknesses:

The authors have used legacy models for all three LLMs, and the study would benefit from testing the current versions of the LLMs (ChatGPT 5.2, Claude 4.5 and Gemini 2.5). Additional metrics such as node coverage, node invention, direction accuracy and sign accuracy would be useful to make robust comparisons across models.

# Author comment: Author response

**DOI:** 10.7554/eLife.109709.3.sa3

**Authors:** Jeevan Tewari, Benjamin W Dahl, B Adam Bates, Jason A Papin, Jeffrey J Saucerman

**Affiliations:** University of Virginia Charlottesville United States; University of Virginia Charlottesville United States; University of Virginia Charlottesville United States; University of Virginia Charlottesville United States; University of Virginia Charlottesville United States

The following is the authors’ response to the original reviews.

**Reviewer #1 (Public review):**

Summary

Large language models (LLMs) have been developed rapidly in recent years and are already contributing to progress across scientific fields. The manuscript tries to address a specific question: whether LLMs can accurately infer signaling networks from gene lists. However, the evaluation is inadequate due to four major weaknesses described below. Despite these limitations, the authors conclude that current general-purpose LLMs lack adequate accuracy, which is already widely recognized. Its key contribution should instead be to provide concrete recommendations for the development of specialized LLMs for this task, which is completely absent. Developing such specific LLMs would be highly valuable, as they could substantially reduce the time required by researchers to analyze signaling networks.

Strengths

The manuscript raises a good question: whether current LLMs can accurately generate signaling networks from gene lists.

Weaknesses:

(1) The authors evaluate LLM performance using only three signaling networks: "hypertrophy", "fibroblast", and "mechanosignaling". Given the large number of well-established signaling pathways available, this is not a comprehensive assessment. Moreover, the analysis need not be restricted to signaling networks. Other network types, including metabolic and transcriptional regulatory networks, are already accessible in well-known databases such as KEGG, Reactome, BioCyc, WikiPathways, and Pathway Commons. Including these additional networks would substantially strengthen the evaluation.

We agree with the reviewer that our evaluation of LLM performance is not comprehensive of all signaling networks, and that the benchmarking was previously limited to signaling networks. The purpose of this study is to benchmark LLMs against peer-reviewed computational models that make testable predictions and are highly validated experimentally, of which these three signaling networks are strong examples. KEGG, Reactome, Biocyc, WikiPathways, and Pathway Commons are databases that contain collections of individual reactions or pathways but are not computational models themselves and have not been experimentally validated in that sense.

While this study focuses primarily on signaling networks, we agree that it would be useful to evaluate how well LLMs perform in generating networks of another type, for which predictive and validated computational models are available. Therefore, in new Figure 3 we now test the ability of LLMs to reconstruct the *E. coli* core metabolic network, as well as test its ability to predict growth on metabolic substrates using flux balance analysis. We find that Claude Opus 4.6, GPT 5.2 Pro, and Gemini 3 Pro Preview perform well at reconstructing reactions from *E. coli* core metabolism, but these reactions are not sufficient to predict growth on a variety of substrates. Given this expansion of scope, we replaced “signaling networks” in the title with “biochemical networks”.

(2) In LLM evaluation, the authors use the gene lists that exactly match those in their "ground truth" networks, thereby fixing the set of nodes and evaluating only the predicted edges. However, in practical research, the relevant genes or nodes are not fully known. A more realistic assessment would therefore include gene lists with both genes present in the ground-truth network and additional genes absent from it, to evaluate the ability of the LLM to exclude irrelevant genes.

We agree with the reviewer that evaluating the capacity of these LLMs to exclude additional genes is interesting. But because biological networks are always incompletely known, there is no “ground truth” of genes absent from a given network. Therefore, for the most rigorous benchmarking against a “ground truth”, we examine the positive predictive ability of LLMs. However, in response to this comment and point 3 below, we further examine additional measures of performance that include “false positives”.

(3) The authors report only the recall/sensitivity of the LLM, without assessing specificity. In practical applications, if an LLM generates a large number of incorrect interactions that greatly exceed the correct ones, researchers may be misled or may lose confidence in the LLM output. Therefore, a comprehensive evaluation must include both sensitivity and specificity. Furthermore, it would be informative to check whether some of the "false positives" might in fact represent biologically plausible interactions that are absent from the manually curated "ground truth". Manually generated "ground truth" can overlook genuine interactions, and the ability of LLMs to recover such missing edges could be particularly valuable. This may even represent one of the most important potential contributions of LLMs.

We agree with the reviewer that additional metrics could inform the evaluation of LLM performance. Therefore, as recommended, we calculated sensitivity, specificity, precision, negative predictive value (NPV), accuracy, and F1 score for each of the network models (hypertrophy, fibroblast, and mechanosignaling). These new results are summarized in confusion matrices shown in a new Supplementary Figures 3, 4, and 5. We performed this additional benchmarking across the 10 replicates for each LLM.

One limitation of this approach is the substantial class imbalance within these confusion matrices. Because we interpret actual negatives as connections that are not found between any nodes of the ground truth models, there will be >10x more true negatives than any of the other classes. This makes specificity, accuracy, and the negative predictive value less informative.

To illustrate this point, consider the ground-truth hypertrophy network which contains 191 connections between 106 nodes. The total number of possible connections between any two nodes is 106^2^ = 11,236. Given that there are 191 actual positives, that leaves 11,045, actual negatives (as illustrated in the null predictor of Supplementary Figure 3A). As the number of node-to-node connections predicted by LLMs is on the order of a few hundred, the number of true negatives is always in the thousands, often outweighing the true positives in the specificity or accuracy calculations.

The effects of this class imbalance are illustrated with the results of the “null predictors” in Supplementary Figure 4A, which are hypothetical models that fail to predict any connection between nodes (have predicted positive values of 0). These null predictors have sensitivities of 0 and specificities of 1 and high accuracies and NPVs because of the high true negative rates.

The precision and F1 scores calculated using these confusion matrices are robust to these class imbalances. Indeed, there is substantial heterogeneity in the number of “false positives” connections generated by the LLMs as illustrated by the precision and F1 scores. We are hesitant to unequivocally label these novel, predicted connections as false positives because, as the reviewer points out, these connections could represent true molecular interactions that were undiscovered at the time of the ground truth models’ conception but have since been demonstrated experimentally and published.

(4) It is widely known that applying differential equation models to highly complex biological networks, such as the three networks in the manuscript, is meaningless, because these systems involve a large number of parameters whose values can drastically alter the results. As Richard Feynman once said: "with four parameters I can fit an elephant, and with five I can make him wiggle his trunk." Thus, the evaluation of LLMs on "logic-based differential equation models" does not make much sense.

Differential equation models have been the primary mathematical framework for studying complex systems for decades. We refer readers unfamiliar with differential equations to the Nobel prize-winning work of Hodgkin/Huxley (Physiology or Medicine1963, action potential of neurons), Prigogine (Chemistry 1977, non-equilibrium thermodynamics and pattern formation), John Nash (Economics 1994, game theory and Nash Equilibrium), Merton/Scholes (Economics 1997, dynamics of financial derivatives), and Manabe/Hasselmann (Physics 2021, dynamic modeling of atmosphere and oceans).

We are confused by the quote of a joke by Richard Feynman about fitting equations to data in the shape of an elephant. While this famous joke is amusing, it is both misattributed (it was a recollection by Enrico Fermi in 1953 of a joke once made by John von Neumann) and deliberately hyperbolic (see https://en.wikipedia.org/wiki/Von_Neumann%27s_elephant). Regardless, the relevance of this joke to our study is unclear, because we are not fitting equations to data. As described in the text, in previous studies we validated the predictions of these three logic-based network models with experimental data that was not used to develop the models.

**Reviewer #1 (Recommendations for the authors):**

(1) All figures are in very poor resolution.

Thank you for identifying this. We have fixed this issue, which was due to SVG embedding. We now embed as higher resolution PNG and provide full resolution files separately.

(2) The manuscript does not include data availability or code availability.

As described in the Methods, all code and data is now available via GitHub (https://github.com/saucermanlab/LLM-network-generation).

**Reviewer #2 (Public review):**

(1) Information on the accuracy of directionality of interaction would help understand if there is a bias towards either a positive or negative association.

To assess if LLMs are biased in predicting either stimulatory or inhibitory connections, we examined the proportion of stimulatory and inhibitory connections for each ground truth model along with the prediction sets from the different LLMs (Author response table 1). These findings suggest that any directional bias is minimal and not conserved across the different ground truth models. These tables were not included in the revised manuscript.

**Author response table 1.** Proportion of stimulatory and inhibitory connections present in each ground truth model and in the sets of connections predicted by each LLM (Claude, GPT, and Gemini).

Hypertrophy	stimulation	inhibition
Ground Truth	86.39	13.61
Claude	91.69	8.31
GPT	89.31	10.69
Gemini	90.54	9.46

The primary subset of reaction types that the LLM’s tend to miss are often downstream, cell-type specific, and/or gene regulatory connection (e.g. Figure 1B). This observation is consistent with the fact that the ground truth models were constructed using experimental evidence from specific publications involving defined experimental models and cell types whereas the LLM’s presumably draw from the entire corpus of published literature.

(2) Do all LLMs capture similar information, or are some LLMs better at capturing certain information than others? Further to this, it would be interesting to look into whether amalgamating information across all three LLMs results in a more accurate network.

The reviewer asks interesting questions that can be qualitatively answered in Figure 1B and in the network visualizations (Supplementary Figures 1 and 2). These diagrams illustrate predicted connections that are shared between the nodes. To include a more quantitative, comprehensive assessment of this overlap, we have included Venn diagrams showing the extent to which LLMs capture shared information (Supplementary Figures 3-5). One such Venn diagram for the hypertrophy model is included in Supplementary Figure 3C. Indeed, it seems that there is substantial overlap in the “false positive” connections predicted by Claude and GPT. It could be interesting to evaluate if these connections are reflective of newly discovered molecular interactions, as referenced in our response to reviewer one point 3.

While amalgamating information across all three LLMs might generate a more accurate network, these Venn diagrams illustrate that there remain connections within the ground truth models that are not represented in any of the prediction sets from the LLMs. Indeed, the union of all predicted connections for the hypertrophy network made by any of the 10 replicates from the different LLMs would still lack 33 ground truth connections (Supplemental Figure 3C).

(3) Would it be possible to retrieve a confidence value of the interactions from the LLMs and conduct Precision, recall rate, AUPR and calibration analyses? These metrics would also help with the benchmarking process.

We agree with the reviewer that including additional evaluative metrics is instructive. Therefore, for each reference network, we have included precision, specificity, recall, accuracy, and F1 score calculations (Supplementary Figures 3-5). Additionally, we conducted calibration analyses to illustrate the LLM’s reliability. While this latter analysis is interesting, we note that the calibration curves were generated using the frequency of predictions among the 10 replicates as a proxy for confidence. This frequency is highly sensitive to the temperature parameter of the LLMs. Different temperature settings could substantially influence the trajectories of these confidence curves and the distributions of the associated histograms. See Supplemental Figure 3B for calibration analyses of the hypertrophy model.

We considered performing analyses resembling AUPR as suggested by the reviewer, but we did see a defensible way to vary a “threshold” for the classification of a connection as positive or negative. In this study, a connection predicted by the LLM either does or does not exist within the ground truth mode.

Typos:

(1) Generated networks to predict THE CLASSIC "fetal gene program" gene expression.

Thank you for catching this error. We have corrected it.

(2) A manually curated network has A functional accuracy of

Thank you for catching this error. We have corrected it.
